# Bisphenol-A plasma levels are related to inflammatory markers, visceral obesity and insulin-resistance: a cross-sectional study on adult male population

**DOI:** 10.1186/s12967-015-0532-y

**Published:** 2015-05-29

**Authors:** Silvia Savastano, Giovanni Tarantino, Vittoria D’Esposito, Federica Passaretti, Serena Cabaro, Antonietta Liotti, Domenico Liguoro, Giuseppe Perruolo, Fabiana Ariemma, Carmine Finelli, Francesco Beguinot, Pietro Formisano, Rossella Valentino

**Affiliations:** Department of Clinical Medicine and Surgery, “Federico II” University of Naples, via Pansini, 5, 80131 Naples, Italy; INT “Fondazione Pascale”, Cancer Research Center of Mercogliano, 83013 Mercogliano, AV Italy; Department of Translational Medical Sciences, “Federico II” University of Naples, via Pansini, 5, 80131 Naples, Italy; Institute of Experimental Endocrinology and Oncology (IEOS), National Council of Research (CNR), “Federico II” University of Naples, via Pansini, 5, 80131 Naples, Italy; Center of Obesity and Eating Disorders, Stella Maris Mediterraneum Foundation, C/da S. Lucia, Chiaromonte, Potenza Italy

**Keywords:** Endocrine-disruptors, Bisphenol-A, Obesity, Visceral adiposity, Metabolic Syndrome

## Abstract

**Background:**

The current increase of obesity and metabolic syndrome (MS) focuses attention on bisphenol-A (BPA), “obesogen” endocrine disruptor, main plastic component. Aim was to verify the role of BPA in metabolic alterations, insulin resistance, low grade inflammation and visceral obesity.

**Methods:**

A cross-sectional study was performed in 76 out of 139 environmentally exposed adult males, unselected Caucasian subjects, enrolled by routine health survey at the “Federico II” University of Naples outpatient facilities. BPA plasma levels (ELISA), metabolic risk factors, homeostasis model assessment of insulin resistance index, plasma monocyte chemoattractant protein 1, interleukin-6 (IL-6) and tumor necrosis factor-alpha were performed. Clinical and biochemical parameters have been compared with BPA and pro-inflammatory cytokines levels.

**Results:**

In total 24 subjects out of 76 (32%) presented with waist circumference (WC) >102 cm, 36 (47%) had impaired fasting glucose and 24 (32%) subjects had insulin resistance [11 out 52 (21%) with WC ≤102 cm and 13 out of 24 with WC >102 cm (54%), χ^2^ 6.825, *p* = 0.009]. BPA and pro-inflammatory cytokine levels were significantly higher in subjects with visceral adiposity (WC > 102 cm). BPA correlated with WC, triglycerides, glucose homeostasis and inflammatory markers. At the multivariate analysis WC and IL-6 remained the main predictors of BPA.

**Conclusions:**

Detectable BPA plasma levels have been found also in our population. The strictly association between BPA and WC, components of MS, and inflammatory markers, further supports the BPA role in visceral obesity-related low grade chronic inflammation.

## Background

Obesity epidemics are responsible for the enormous strains on our health-care system, also concerning economical aspects. It has been proposed that the rapid increase in the environment of endocrine disrupting compounds (EDCs), in particular bisphenol-A (BPA), can be responsible for energy homeostasis disorders [1–3]. In this respect, several studies, mainly in animals, evidenced the pathogenetic role of BPA in modifying genes involved in obesity in impacting on visceral adipose tissues function, both processes ending up in metabolic syndrome (MS) and cardio-metabolic diseases [3–5].

BPA, as xenoestrogen lipophilic compound, accumulates into adipose tissue [6]. As major plastic component, BPA causes a widespread environmental human exposure via food and beverage containers, dental composites, thermal paper and airborne dust. Moreover, it is able to migrate into food and water, mainly upon heating [3, 5, 7–10]. BPA is present in body fluids of the normal population. In particular, the 2003–2004 NHANES III conducted by the CDC, found detectable levels of BPA in 93% of 2,517 urine samples from people 6 years and older [11–13]. Nevertheless, the association between urinary BPA and adverse health outcomes could be affected by the short half-life of BPA and the presence of conjugate and inactive forms of BPA in urinary specimens [14–17]. On the other hand, gender differences in serum/plasma BPA concentrations, possibly due to differences in the androgen-related metabolism of BPA, are well documented, with increase in the androgen-related glucuronidation by liver microsomes [18]. Although the BPA exposure is decreasing from consumer merchandise and by the substitution with bisphenol-S, an analog of BPA used in BPA-free products, is increasingly, BPA-free products containing BPS are not necessarily safer [19].

A number of studies investigated the possible association between BPA exposure and adverse health outcomes. Indeed, this chemical compound may be involved in adipose tissue dysfunction, but also in metabolic/endocrine dysfunctions, cancer and fertility problems [11–16]. As collateral finding, a positive association between higher levels of urinary BPA and MS parameters, impaired plasma glucose has been recently reported [12, 15], such as its involvement in insulin resistance, particularly in animal models exposed during fetal life [20, 21]. BPA plasma levels may be considered a reliable method to measure environment-linked chronic exposure [8, 16, 17] avoiding interference due to gender and age-relate individual differences [18, 22]. Although urine measurement is considered the best and easiest way to evaluate BPA, a spot of urine sample may not be appropriate to evaluate the real continuous chronic exposure, because largely affected by food consumption [7–10, 16–18]. In addition, in urine BPA is unstable and mainly in its conjugated and inactive form, evaluating only food and beverage contamination and excluding non-oral routes of exposure, which avoid the first pass of hepatic metabolism [22–24]. Finally, a clear effect of time of exposure and age-dependent differences in metabolic clearance might be responsible for different effects of BPA on human health [16, 17, 25]. On the ground of this evidence, we have implanted a study in an adult male population living in southern Italy with the aim to investigate the association between plasma BPA levels and both components of the MS and markers of inflammation, considering that the low grade chronic inflammation has emerged as a key pathogenic link between visceral adiposity, insulin resistance (IR) and MS [26].

## Methods

### Subjects

A cross-sectional study was conducted on 76 males out of 139 unselected Caucasian subjects of both gender undergoing a routine health survey at the “Federico II” University Hospital of Naples outpatient facilities. All the subjects belonged to the workforce of the municipality of Naples (Italy), representative of the general Italian population living in southern Italy, were consecutively recruited. The study protocol was approved by the Ethic Committee “Carlo Romano” of “Federico II” University of Naples and conducted in accordance to the principles of the Declaration of Helsinki as revised in 2000. The purpose of the protocol was explained to all participants and written consent was obtained at the beginning of the study. Exclusion criteria were: (1) female gender due to possible interaction of BPA-by-sex; (2) type 2 diabetes (T2D) according to the American Diabetes Association; (3) renal failure, advanced liver diseases, cancer and acute viral, bacterial or fungal infection; (4) acute or chronic inflammatory diseases; (5) pharmacological treatments potentially impacting on inflammation or acting on gluco-lipids metabolism, or appetite modifiers, including vitamins and anti-oxidant agents. A balanced antihypertensive regimen was maintained for those suffering from hypertension through the study.

Age, race/ethnicity, eating habits, physical activity, smoking status, alcohol intake (grams per day), level of education and physical activity were assessed using a self-made written questionnaire, such as the detailed medical history (including information on T2D-affected relatives and cardiovascular risk).

History, clinical data and blood samples were simultaneously collected.

Systolic (SBP) and diastolic (DBP) blood pressure were measured in the left arm of the patient, after 5 min of quiet rest, using a mercury sphygmomanometer according to the American Heart Association recommendations. Up to three measurements were averaged for SBP and DBP.

### Anthropometric measurements

All anthropometric measurements were taken with subjects wearing only light clothes and without shoes. Height was measured to the nearest centimeter using a wall-mounted stadiometer. Body weight was determined to the nearest 50 g using a calibrated balance beam scale. Body mass index (BMI) was calculated as weight (kg)/height squared (m^2^) using the following cut off points: normal weight 18.5–24.9, overweight 25–29.9, class I obesity 30–34.9. WC, as index of visceral adiposity, was measured using a flexible steel tape at the natural indentation or at a level midway between the iliac crest and the lower edge of the rib cage if no natural indentation was visible.

### Blood samples

For each subject, whole-blood samples were drawn from an ante-cubital vein in the morning after an overnight fast on two separate occasions. All plasma samples collected were divided in aliquots of 500 µl and transferred into a clean polypropylene tube, and then stored at −80°C until analysis. Total, high-density lipoprotein (HDL) and low-density lipoprotein (LDL) cholesterol and triglycerides (TG) levels were assayed in a centralized facility, using commercial methods. Fasting plasma glucose (FPG) levels were determined by the glucose oxidase method. Insulin ELISA was measured by solid-phase two-site enzyme immunoassay (Mercodia AB, Sylveniusgatan 8A SE-754 50 Uppsala-Sweden). The intra-assay and inter-assay coefficients of variation were less than 4 and 3.6%, respectively.

The homeostasis model assessment index of insulin resistance (HoMA-IR) was calculated according to the formula [fasting glucose (mmol/l) × fasting insulin (mU/l)/22.5], as previously reported, and HoMA-IR >2.5 was considered to be diagnostic for IR [27, 28].

### Metabolic risk factor assessment

Metabolic risk factors were defined according to The National Cholesterol Education Program’s Adults Treatment Panel III (NCEP-ATP III) revised criteria [29], i.e. WC > 102 cm; TG > 150 mg/dl; HDL < 40 mg/dl; SBP > 130 mmHg or DBP > 85 mmHg and fasting plasma glucose >100 mg/dl.

### Markers of inflammation

Plasma monocyte chemoattractant protein 1 (MCP-1), interleukin-6 (IL-6) and tumor necrosis factor-alpha (TNFα) were measured using an Human BIO-PLEX Suspension Array Multi-panel System (BIORAD Laboratories, Inc.), according to the manufacturer’s protocol.

### BPA assay

Total plasma concentrations of BPA (free and conjugated) were assayed with a kit from IBL Co. (Ltd., Gunma, Japan), based on a competitive ELISA protocol, using the anti-rabbit IgG antibody solid-phase method and characterized by a measurement range of 0.3–100 ng/ml BPA. To better sensitize this ELISA method on plasma samples, we have modified the standard curve, obtaining a BPA low limit of detection of 0.025 ng/ml. The cross reactivity for total BPA was 100%, for BPA-Glucuronide 85%, for BPA–Na-Sulphate 68%, for Bisphenol B 8.3%, and for other related compounds less that 0.02%. The intra- and inter-assay coefficients of variation (CV) were <14 and 5%, respectively. All standards and samples were measured in duplicate. As low and high internal control, we used a control plasma added with BPA 1 and 25 ng/ml, respectively.

### Statistical analysis

Results are expressed as mean ± SD or as median plus range according to variable distributions, as indicated. Differences in variables between groups were analyzed using the unpaired Student *t* test or Mann–Whitney *U* test, when appropriate. Pearson r or Spearman’s rho correlation coefficients were used to analyze the association between variables. Frequencies among groups were analyzed by χ^2^ for trend. In suspicion of heteroscedasticity, i.e., if there are sub-populations that have different variability from others in the homoscedastic model and having detected the presence of few outliers, to avoid a dramatic increase in the type II error rate, we analyzed the correlation between BPA and WC by the robust regression. The method for estimating the parameters (coefficient, standard error and 95% confidence intervals, CI) in a linear regression model was ordinary least squares (OLS) [30]. Using BPA as dependent variable, a multiple linear regression analysis model was performed with the enter selection methods: in this model WC, HoMA-IR, IL-6, and TNFα were included as independent variables. A *t*-stat >1.96, with a significance <0.05, indicates that the independent variable is a significant predictor of the dependent variable within and beyond the sample. To avoid multicollinearity, i.e. situations in which the predictors are correlated to each other to some degree, the variance inflation factor and tolerance were set to >10 and <0.1, respectively. *P* value <0.05 was considered statistically significant. Packages used for running statistics were SYSTAT 13 (2009) and MedCalc 14.8.1 (2014).

## Results

### Subjects: clinical and metabolic parameters

Clinical and biochemical markers of the study population was reported in Table 1. According to BMI cut off points, 36 subjects presented with normal weight, 28 were overweight and 12 had class I obesity.

**Table 1:** Clinical and biochemical parameters of the study population

Parameters	Values
Age (years)	53.5 ± 5.7
BMI (kg/m^2^)	25.7 ± 3.4
WC (cm)	96.8 ± 10.1
SBP (mmHg)	120 (90–160)
DBP (mmHg)	80 (60–100)
FPG (mg/dl)	100.9 ± 11.8
Insulin (mU/l)	6.1 (1.2–28.7)
HoMA-IR	1.4 (0.4–8.4)
Total cholesterol (mg/dl)	208.1 ± 46.1
HDL (mg/dl)	49.8 ± 11.7
LDL (mg/dl)	129.8 ± 42.1
TG (mg/dl)	154.6 ± 73.6
BPA (ng/ml)	1.04 ± 0.77
IL-6 (pg/ml)	0.77 (0.06–3.43)
MCP-1 (pg/ml)	27.4 ± 23.5
TNFα (pg/ml)	1.9 (0.5–9.1)

Data are expressed as mean ± standard deviation or median, according to variable distribution. In particular, SBP, DBP, insulin, HoMA-IR, IL-6 and TNFα were expressed an median with range.

*BMI* body mass index, *WC* waist circumference, *SBP* systolic blood pressure, *DBP* diastolic blood pressure, *FPG* fasting plasma glucose, *HoMA-IR* homeostasis model of assessment-insulin resistance, *HDL* high-density lipoprotein, *LDL* low-density lipoprotein, *TG* triglycerides, *BPA* bisphenol-A, *IL-6* interleukin-6, *MCP-1* monocyte chemoattractant protein-1, *TNFα* tumor necrosis factor-alpha.

**Figure 1:**
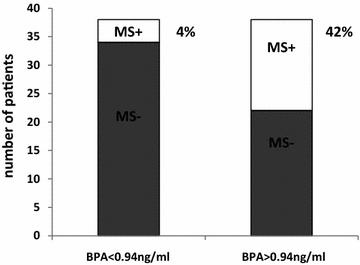
Percentage of patients affected by metabolic syndrome (MS) in the two groups divided considering the median bisphenol-A (BPA) plasma value of 0.94 ng/ml. Four subjects out of 38 (11%) in the group with BPA plasma levels <0.94 ng/ml have MS, while 16 out of 38 (42%) were MS positive (MS+) in the group with BPA plasma levels >0.94 ng/ml.

In the whole population, 24 subjects (32%) presented with visceral adiposity (WC > 102 cm), while 34 (45%) and 27 (36%) subjects had increased SBP and DBP, respectively. Forty subjects (53%) were euglycemic and the remaining 36 had impaired fasting glucose, while values of HoMA-IR >2.5 were found in 24 subjects (32%). The minimum and maximum BPA plasma values of the our study population were 0.04 and 3.3 ng/ml, respectively, with a mean value in line with other population studies. In particular, considering the median BPA value (0.94 ng/ml), the diagnosis of MS was made in four subjects (11%) with low BPA *vs* 16 (42%) with high BPA (χ^2^ 8.211; p = 0.0042) (Figure 1).

Dividing the population according to WC (Table 2), there were significant differences in BMI, HDL, insulin, HoMA-IR, as expected. In particular, 11 subjects out of 52 (21%) with WC ≤ 102 cm and 13 out of 24 with WC > 102 cm (54%) were insulin-resistant (χ^2^ 6.825; p = 0.009). In addition, BPA (*p* = 0.002), IL-6 (*p* = 0.030) and TNFα (*p* = 0.007) levels were significantly higher in the subjects with WC >102 cm.

**Table 2:** Clinical and biochemical parameters of the study population according to waist circumference

Parameters	WC ≤ 102 cm (n. 52)	WC > 102 cm (n. 24)	*p* values
Age (years)	53.6 ± 6.0	53.4 ± 5.2	0.900
BMI (kg/m^2^)	24.0 ± 2.5	29.1 ± 2.5	<0.0001
SBP (mmHg)	122.5 (90–160)	120.0 (100–160)	0.819
DBP (mmHg)	80.0 (65–95)	80.0 (60–100)	0.305
FPG (mg/dl)	101.4 ± 12	99.9 ± 11.7	0.613
Insulin (mU/l)	3.6 (1.2–26.4)	10.4 (3.3–28.7)	0.0006
HoMA-IR	0.9 (0.4–7.7)	2.7 (0.7–8.4)	0.0016
Total cholesterol (mg/dl)	212.0 ± 50.1	199.5 ± 35.2	0.273
HDL (mg/dl)	52.7 ± 11.8	43.6 ± 9.1	0.001
LDL (mg/dl)	132.9 ± 46.5	123.0 ± 30.2	0.344
TG (mg/dl)	149.8 ± 81.5	165.0 ± 53.4	0.407
BPA (ng/ml)	0.86 ± 0.72	1.44 ± 0.74	0.002
IL-6 (pg/ml)	0.77 (0.06–2.2)	0.78 (0.07–3.43)	0.030
MCP-1 (pg/ml)	26.9 ± 25.4	28.3 ± 19.3	0.808
TNFα (pg/ml)	1.1 (0.5–9.1)	3.2 (0.6–8.3)	0.007

Data are expressed as mean ± standard deviation or median, according to variable distribution. In particular, SBP, DBP, Insulin, HoMA-IR, IL-6 and TNFα were expressed as median with range. Differences in variables between groups were analyzed using the unpaired Student *t* test or Mann-Whitney *U* test, when appropriate.

*BMI* body mass index, *WC* waist circumference, *SBP* systolic blood pressure, *DBP* diastolic blood pressure, *FPG* fasting plasma glucose, *HoMA-IR* homeostasis model of assessment-insulin resistance, *HDL* high-density lipoprotein, *LDL* low-density lipoprotein, *TG* triglycerides, *BPA* bisphenol-A, *IL-6* interleukin-6, *MCP-1* monocyte chemoattractant protein-1, *TNFα* tumor necrosis factor-alpha.

### Correlation studies

**Figure 2:**
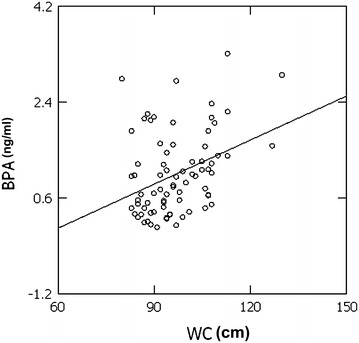
The correlation between bisphenol-A (BPA ng/ml) and waist circumference (WC cm) was analyzed by the robust regression. A strict prediction by BPA towards WC was found.

Next, we searched for correlations between BPA plasma levels and clinical and metabolic parameters in the whole study population (Table 3). BPA levels were significantly correlated with WC, TG, and markers of glucose homeostasis (FPG, insulin, HoMA-IR). In addition, significant correlations were found between BPA and inflammatory markers, such as IL-6 and TNFα. At robust regression, a strict prediction by BPA towards WC was found with the following results: coefficient 0.033, standard error 0.008, CI 0.017–0.048 (Figure 2). At the multivariate analysis WC and IL-6 remained the main predictors of BPA (Table 4).

**Table 3:** Correlations between BPA and clinical and metabolic parameters

Parameters	*r*	*p*
Age	0.072	0.535
BMI	0.187	0.106
WC	*0.363*	*0.001*
SBP	0.174	0.132
DBP	0.154	0.184
FPG	*0.338*	*0.003*
Insulin	*0.256*	*0.025*
HoMA-IR	*0.268*	*0.019*
Total cholesterol	0.040	0.731
HDL	−0.181	0.117
LDL	−0.008	0.949
TG	*0.275*	*0.016*
IL-6	*0.320*	*0.005*
MCP-1	−0.166	0.152
TNFα	*0.248*	*0.031*

Pearson r or Spearman’s *rho* correlation coefficients were used to analyze the association between variables.

Italic *r* values are Pearson *r* or Spearman’s *rho* correlation coefficients, when appropriate. Italic *p* values are statistically significant.

*BMI* body mass index, *WC* waist circumference, *SBP* systolic blood pressure, *DBP* diastolic blood pressure, *FPG* fasting plasma glucose, *HoMA-IR* homeostasis model of assessment-insulin resistance, *HDL* high-density lipoprotein, *LDL* low-density lipoprotein, *TG* triglycerides, *BPA* bisphenol-A, *IL-6* interleukin-6, *MCP-1* monocyte chemoattractant protein-1, *TNFα* tumor necrosis factor-alpha.

**Table 4:** Multivariate analysis

Parameters	β	*t*	*p*
WC	0.298	2.674	0.008
IL-6	0.237	2.123	0.037

Using bisphenol-A as dependent variable, a multiple linear regression analysis model was performed with the enter selection methods: in this model WC, HoMA-IR, IL-6, and TNFα were included as independent variables. Variable excluded: HoMA-IR and TNFα.

*WC* waist circumference, *IL*-*6* interleukin-6.

## Discussion

In the present study we have obtained evidence that the adult males living in Southern Italy have detectable BPA plasma levels. In addition, we found that higher BPA plasma levels were associated with higher WC and TG values and with alteration in glucose homeostasis. In this respect, our data are generally confirmatory of the literature on the wide diffusion of the BPA environmental exposure among different populations and on the association of this exposure with the MS and visceral adiposity in humans [4, 8, 12, 13, 15]. Furthermore, higher BPA levels were associated with higher circulating IL-6 and TNFα levels, usually considered as useful markers of low grade inflammation related to visceral adiposity. MCP-1, marker of chronic inflammation involved in endothelial dysfunction, hypertension and cardiovascular risk in obese subjects, did not changed. Although the statistically significant difference in circulating cytokines not necessarily bears a biological significance, to the best of our knowledge this study reports the first evidence of the association between BPA plasma levels, markers of visceral adiposity dysfunction and inflammation in adult males. Previous studies investigated the association between BPA and altered glucose homeostasis [7, 8, 11, 14, 15]. The strong correlation between BPA, WC, IL-6 and TNFα plasma levels supports the pathogenetic involvement of BPA in increasing visceral adiposity and determining low grade chronic inflammation.

Of interest, in our study visceral adiposity and inflammation, but not HoMA-IR, were the main predictors of BPA plasma levels. This association let us to speculate that the well-known correlation between altered glucose homeostasis and BPA could be only the epiphenomenon of the association between BPA and visceral adiposity dysfunction, with inflammation as marker of this association. This evidence further support the hypothesis that in individuals with BPA environmental exposure, the derangement of glucose metabolism and inflammatory pathways might be associated independently from the inflammatory pathways induced by visceral adiposity *per se*. In line with data by Teppala et al. [12] obtained in a representative sample of US adults, we also found that higher prevalence of MS occurred in subjects with higher BPA plasma levels. As support for the direct involvement of BPA in inflammatory and dysmetabolic processes in vivo, we have recently obtained evidence in vitro on adipocyte cultured with BPA doses (1 nM = 23 ng/ml) considered low in cultured cells, but in the range of typical human exposure. These adipocytes displayed an activation of inflammatory pathways and alteration of insulin signaling [31]. However, additional in vitro experimental studies, particularly on adipogenesis, are necessary in order to unravel how BPA continuous environmental exposure can act on adipose tissue differentiation with subsequent dysfunction and alteration in glucose and lipid homeostasis, affecting human metabolic health.

Concerning human BPA exposure, we have also demonstrated that in women with polycystic ovary syndrome (PCOS), a condition typically characterized by IR and low grade chronic inflammation, the association between BPA plasma levels and markers of low grade inflammation, in particular spleen size, a reliable and a stable index of chronic inflammation, occurred independently of obesity. In the same study, higher BPA levels identified a subgroup of PCOS women characterized by higher markers of low grade chronic inflammation, increased prevalence of hepatic steatosis, and more severe IR and hyperandrogenism [32].

We are aware that the main limitation of this study is the relatively small sample size for a cross sectional study, but it was not simple to acquire a large homogenous cohort in short time, also because we have included only environmentally exposed adult males from Caucasian unselected homogeneous population. This aspect suggests that future studies with larger sample size are needed. However, the strength of this study was the homogeneity of the population sample, including only adult male participants of the same ethnic group, geographic area and occupancy, to avoid the confounding effects on gender, race, time and modality of environmental BPA exposure. Furthermore, although BPA plasma levels may be considered a reliable method to measure chronic exposure, there is a recent tendency to prefer urine as the most appropriate and robust marker for BPA exposure assessment.

## Conclusions

The evidence that individuals in the world have measurable plasma levels of BPA is now confirmed also in our adult population living in southern Italy. The subjects with higher BPA plasma values presented markers of low grade inflammation, higher visceral adiposity and higher prevalence of MS and insulin resistance.
